# Comparison of unilateral and bilateral percutaneous vertebroplasty for osteoporotic vertebral compression fractures: a systematic review and meta-analysis

**DOI:** 10.1186/s13018-016-0479-6

**Published:** 2016-12-01

**Authors:** Haolin Sun, Chunde Li

**Affiliations:** Department of Orthopedic Surgery, Peking University First Hospital, No. 8 Xishiku St., Xicheng District, Beijing, 100034 China

**Keywords:** Unilateral percutaneous vertebroplasty, Osteoporotic vertebral compression fractures, Bilateral treatment

## Abstract

**Background:**

The aim of this meta-analysis is to examine the safety and effectiveness of unilateral percutaneous vertebroplasty (PVP) for treatment of osteoporotic vertebral compression fractures (OVCFs) compared with that of bilateral treatment.

**Methods:**

The multiple databases including PubMed, Springer, EMBASE, OVID, and China Journal Full-text Database were adopted to search for relevant studies in English or Chinese, and full-text articles involving comparison of unilateral and bilateral PVP surgery were reviewed. Review Manager 5.0 was adopted to estimate the effects of the results among selected articles. Forest plots, sensitivity analysis, and bias analysis for the articles included were also conducted.

**Results:**

Finally, 1043 patients were included in the 14 studies, which eventually satisfied the eligibility criteria, and unilateral and bilateral surgeries were 550 and 493, respectively. The meta-analysis suggested that there was no significant difference of VAS score, ODI score, and cement leakage rate (MD = 0.12, 95%CI [−0.03, 0.26], *P* = 0.11; MD = −1.28, 95%CI [−3.59, 1.04], *P* = 0.28; RR = 0.89, 95%CI [0.61, 1.29], *P* = 0.52). The surgery time of unilateral PVP is much less than that of bilateral PVP (MD = −16.67, 95%CI [−19.22, −14.12], *P* < 0.00001). Patients with bilateral PVP surgery have been injected more cement than patients with unilateral PVP surgery (MD = −1.55, 95%CI [−1.94, −1.16], *P* < 0.00001).

**Conclusions:**

Both punctures provide excellent pain relief and improvement of life quality. We still encourage the use of the unipedicular approach as the preferred surgical technique for treatment of OVCFs due to less operation time, limited X-ray exposure, and minimal cement introduction and extravasation.

## Background

Osteoporosis is an important health issue in ageing populations, characterized by low bone mass that leads to fragile bones and higher fracture risks [1, 2]. Osteoporosis and associated fractures are the cause of morbidity in older adults, and osteoporotic vertebral compression fracture (OVCF) is the most common one, which affects more than 200 million individuals worldwide [3]. It occurs more frequently than ankle, wrist, or hip fractures, and it may occur spontaneously just in a simple activity such as picking up something or just rising from a chair [4]. Patients with osteoporotic vertebral fractures (OVFs) suffer severe back pain for weeks to months, and spinal deformities, reduced pulmonary function, restriction of the abdominal and thoracic contents, impaired mobility, and clinical depression caused by OVCFs produce effects on patient quality of life [5–8]. OVCFs can affect both the elderly male and female. Studies suggested that OVCFs are developed in 8% of women older than 50 years and 27% of men and women older than 65 years [9, 10].

OVCFs have traditionally been treated with bed rest, analgesic use, physical therapy, and antiresorptive medications. But these treatments are conservative managements which cannot reverse the kyphotic deformities, and also cause comorbidities including deep venous thrombosis, acceleration of osteopenia, respiratory problems, and emotional problems [4, 11]. Besides, because of the poor quality of osteoporotic bone, classical surgery with metal implants often fails and contributes to persistent back pain, neurological symptoms, and functional limitations [8, 12]. Percutaneous vertebroplasty (PVP), which was introduced by Galibert in 1984 for treating osteolytic metastasis, myeloma, and hemangioma [13], is a minimally invasive surgical procedure that has gained popularity as a new treatment of OVCFs. The procedure includes placing spinal needles into fractured vertebral bodies and injecting polymethylmethacrylate (PMMA) or other bone cements into the fractured vertebral body under radiological control to relieve pain and increase bone strength [14]. After the injection of the cement, pain pathways in the surrounding tissue seem to be altered in response to various stimulations. Mechanism stabilization of the fracture, such as thermal injury to the nerve endings, results in immediate pain relief [15]. It has been reported that PVP can produce immediate pain relief compared to conservative treatments [16].

PVP is one of the optimal treatments for OVCFs and provides rapid pain relief and stabilization of fractured vertebral bodies [17, 18], but the matter of surgical approach selection remains controversial. As a minimally invasive technique, the standard technique is typically carried out using the bipedicular approach [19, 20]. Some researchers believe that bilateral PVP is more superior for excellent pain relief, which is associated with symmetrical distribution of bone cement in the vertebral body [15, 21]. But in recent years, unilateral PVP is being increasingly used for the reduction of operation and radiation exposure time and the lower risk of cement leakage and complications [22, 23]. In theory, bilateral PVP shows increased surgery time and injected cement volume, and the risk of bone cement leakage is twice that of the unipedicular approach; on the other hand, unilateral PVP can reduce the operation time, surgery-associated complications, radiation exposure, and cost, and unilateral PVP can also achieve the same clinical results.

The aim of this meta-analysis is to examine the safety and effectiveness of unilateral percutaneous vertebroplasty for treatment of osteoporotic vertebral compression fractures compared with that of bilateral treatment. Owing to the inconsistent results of studies about shear bond strength, it is necessary to perform a systematic review and meta-analysis to study the difference of curative effects and complication rates of surgery between unilateral and bilateral PVP, which will undoubtedly increase surgeon confidence of both medical staffs and patients.

## Methods

### Search strategy

Related citations about unilateral and bilateral PVP surgery were systematically searched, and a systematic review was undertaken with articles published from January 2000 to January 2016 among multiple electronic databases. To assemble all of the relevant published citations, PubMed, Springer, EMBASE, OVID, and China Journal Full-text Database were searched. All publication statuses (published, unpublished, in press, and in progress) were included. We searched the literature independently, and studies were initially reviewed by titles and abstracts. No restrictions about the publication language were made.

The following keywords were used to maximize the search specificity and sensitivity in our search work: (1) osteoporotic vertebral compression fractures OR OVCF OR VCF, (2) percutaneous vertebroplasty OR vertebroplasty OR PVP OR VP, and (3) unilateral OR bilateral OR unipedicular OR bipedicular. MeSH terms and Boolean operators were selected for each database search. All the citations searched out were screened for further selection.

### Citation selection

Both my companion and I selected the citations in this process, independently and attentively. They screened the titles and abstracts of the articles identified by the electronic search criteria presented above. Subsequently, the full text of the studies that potentially met the criteria were obtained and reviewed to check whether the study was likely to be relevant.

These relevant studies included in this study must meet the following inclusion criteria:

1. Adult patients with osteoporotic vertebral fractures
2. Sample size of more than 20
3. A randomized control trial or controlled clinical trial study
4. Comparison between unilateral and bilateral PVP
5. Available exclusion criteria

Exclusion criteria:

1. Non-randomized studies
2. Studies on other diseases rather than OVCFs
3. Studies lacking outcome measures or comparable results

After the primary selection, these two researchers met and reviewed their selections for agreement. Disagreements were resolved by reaching a consensus through discussion.

### Data extraction

Two of the reviewers independently read the full text of the articles and extracted the characteristics from each study using a standard data extraction form in Excel 2010. The data extracted from these studies included the first author’s name, year of publication, year of onset, mean age of patients, sample size (unilateral/bilateral), sex distribution (male/female), outcome measurements, and follow-up time. Outcome measurements, including visual analog scale (VAS), Oswestry Disability Index (ODI), surgery time, injected cement volume, and cement leakage outcome were collected to estimate the difference between unilateral and bilateral PVP.

### Statistical analysis

We performed all of the meta-analyses with Review Manager 5.0 (The Cochrane Collaboration, 2011) to estimate the safety and effectiveness of unilateral PVP compared with that of bilateral treatment among selected articles. Following the Review Manager 5.3 Tutorial, the risk-of-bias table of the included studies was independently assessed by two authors. The assessment included the following criterions: (1) random sequence generation, (2) allocation concealment, (3) blinding of participants and personnel, (4) blinding of outcome assessment, (5) incomplete outcome data, (6) selective reporting, and (7) other biases. Any disagreements were resolved by discussion. If any problems of poor agreement occurred or no consensus could be achieved, a third investigator was the adjudicator.

For continuous outcomes, including VAS, ODI, surgery time, and injected cement volume, standard mean difference (SMD) with 95% confidence intervals (CIs) was calculated by the mean and standard deviation. Related risk (RR) with 95%CIs was calculated to estimate the cement leakage outcome. A *P* value <0.05 was considered to be statistically significant.

Heterogeneity was assessed using *Q* statistics in this study. The value of *I* ^2^ statistic reflects the levels of heterogeneity. A random-effect model was adopted when moderate or high heterogeneity was obtained, which means the heterogeneity *I* ^2^ statistic is >50%; otherwise, a fixed-effect model was chosen.

In addition, sensitivity analysis and bias analysis of the studies were conducted to examine the quality of articles. To estimate possible publication bias, a funnel plot was used.

## Results

### Search results

**Fig. 1:**
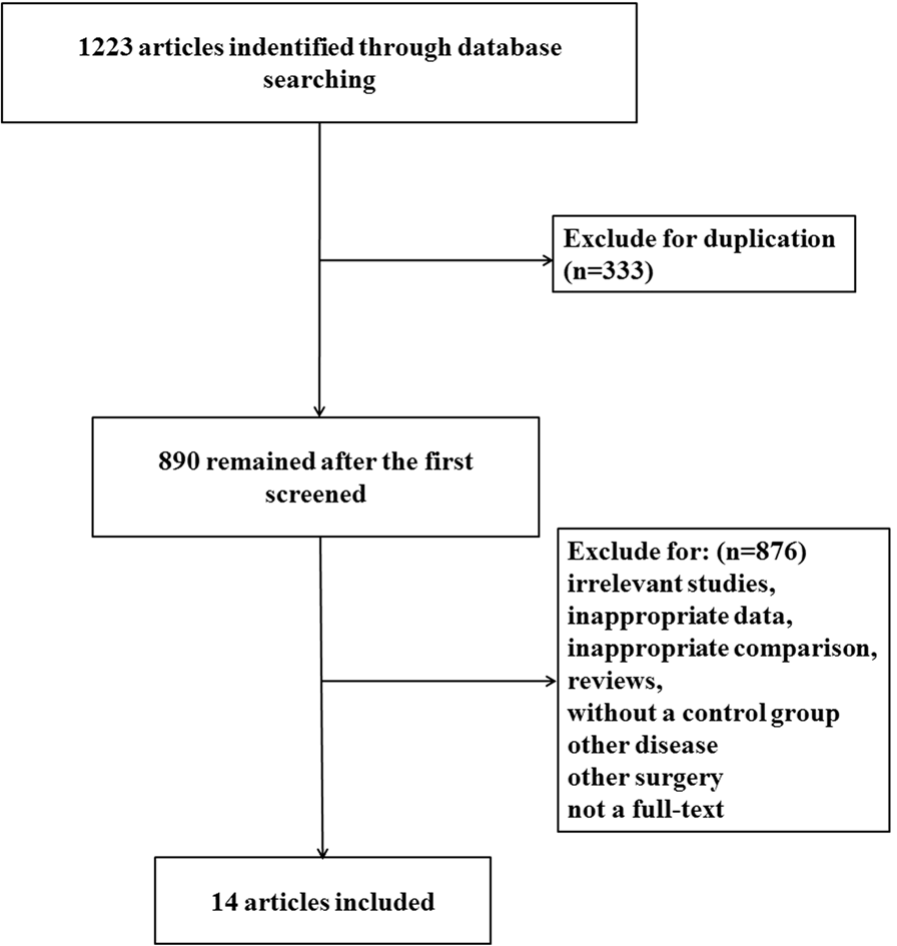
Flow diagram of the study selection showing the number of citations identified, excluded, and included in final analysis

A total of 1223 titles and abstracts were preliminarily reviewed in these electronic databases after the primary selection, of which 14 studies [22, 24–36] eventually satisfied the eligibility criteria. The other 1207 articles were excluded for duplication, irrelevant studies, inappropriate data, inappropriate comparison, reviews, without a control group, other diseases, other surgeries, or not a full text. A flow diagram that reflects the search process can be seen in Fig. 1 including the reasons for exclusion. Among these 14 articles, 9 report on VAS score for analgesic efficacy evaluation, 4 on ODI for functional assessment, 9 on surgery time, 11 on injected cement volume, and 9 on cement leakage.

### Characteristics of included studies

Detailed characteristics of the included studies are provided in Table 1. The first author’s name, year of publication, year of onset, mean age of patients, sample size (unilateral/bilateral), sex distribution (male/female), outcome measurements, and follow-up time are presented in the table. All these articles were published from 2013 to 2015. In total, 1043 patients were included in the 14 studies, and unilateral and bilateral surgeries were 550 and 493, respectively. The sample size ranges from 39 to 153. All patients in these studies were adults.

**Table 1:** Characteristic of the included studies

Author	Year of publication	Year of onset	Mean age	Sample size (unilateral/bilateral)	Sex distribution (male/female)	Outcome measurements	Follow-up time
Chen CM [25]	2014	–	Unilateral group, 69.43 ± 6.25; bilateral group, 68.66 ± 8.76	39 (20/19)	–/–	VAS, ODI, surgery time, Cement leakage, injected cement volume	2 years
Feng Y [26]	2014	February 2010 to February 2012	Unilateral group, 72.5; bilateral group, 69.4	117 (61/56)	30/87	VAS, ODI, injected cement volume	2 years
Guo ZP [27]	2015	March 2008 to March 2014	Unilateral group, 67.32 ± 11.24; bilateral group, 69.35 ± 12.46	153 (91/62)	29/124	VAS, ODI, surgery time, Injected cement volume, cement leakage	–
Li J [28]	2015	April 2007 to February 2014	Unilateral group, 67.9; bilateral group, 65.7	65 (36/29)	30/35	VAS, surgery time, injected cement volume, cement leakage	6 to 18 months
Ren HL [29]	2014	January 2009 to January 2012	Unilateral group, 69.4 ± 10.4; bilateral group, 69.7±9.7	101 (45/56)	17/84	Surgery time, injected cement volume, cement leakage	More than 1 year
Wang W [30]	2013	February 2009 to February 2011	Unilateral group, 66.9; bilateral group, 68.7	47 (25/22)	18/29	VAS, cement leakage	1 year
Xiao L [31]	2015	September 2012 to December 2014	Unilateral group, 70.9 ± 9.3; bilateral group, 68.5±7.3	71 (40/31)	–/–	VAS, injected cement volume, cement leakage	3 days
Yuan WQ [32]	2014	June 2013 to June 2014	70.36 ± 0.35	72 (36/36)	33/39	Surgery time, injected cement volume	2 days
Zhai HL [33]	2013	January 2010 to February 2012	Unilateral group, 70.5; bilateral group, 74.3	48 (27/21)	6/42	VAS, surgery time, injected cement volume	1 day
Zhang L [34]	2015	November 2010 to October 2012	Unilateral group, 71.7 ± 7.5; bilateral group, 72.1 ± 6.0	50 (24/26)	13/37	VAS, ODI, PCS, MCS, surgery time, cement leakage, injected cement volume	2 years
Zhang LG [35]	2015	January 2008 to December 2011	Unilateral group, 70.0 ± 2.9; bilateral group, 70.7 ± 2.5	68 (36/32)	0/68	VAS, QUALEFFO, cement, cement leakage	1 year
Zhang X [36]	2014	March 2012 to August 2013	70 ± 0.27	53 (28/25)	25/28	Surgery time, injected cement volume	–
Zhao XQ [37]	2014	May 2011 to February 2013	Unilateral group, 74; bilateral group, 73	80 (40/40)	48/32	Curative effect, surgery time, injected cement volume	2 days
Zhou R [38]	2015	February 2011 to January 2013	Unilateral group, 68.8 ± 5.1; bilateral group, 70.6 ± 4.8	79 (41/38)	46/33	VAS, surgery time, injected cement volume, cement leakage	1 year

### Quality assessment

All of the non-randomized control trials had insufficient information on the randomization methods. All 14 included studies were grouped randomly, but the methods of randomization were not mentioned. So we classified these articles as controlled clinical trial studies. The risk-of-bias table was used to evaluate the risk of each study. The risk-of-bias table in this meta-analysis is shown in Table 2. High risk of blinding of participants and personnel existed for the particularity of the operation.

**Table 2:** The risk-of-bias table in this meta-analysis

	Chen CM [25]	Feng Y [26]	Guo ZP [27]	Li J [28]	Ren HL [29]	Wang W [30]	Xiao L [31]	Yuan WQ [32]	Zhai HL [33]	Zhang L [34]	Zhang LG [35]	Zhang X [36]	Zhao XQ [37]	Zhou R [38]
Random sequence generation	Low	Low	Low	Low	Low	Low	Low	Low	Low	Low	Low	Low	Low	Low
Allocation concealment	Not	Not	Not	Not	Not	Not	Not	Not	Not	Not	Not	Not	Not	Not
Blinding of participants and personnel	High	High	High	High	High	High	High	High	High	High	High	High	High	High
Blinding of outcome assessment	Low	Low	Low	Not	Low	High	Low	High	Low	Low	Low	Low	High	High
Incomplete outcome data	Low	Low	Low	Low	Low	Low	Low	Low	Low	Low	Low	Low	Low	Low
Selective reporting	Not	Low	Not	High	Not	High	Low	Low	Low	Low	Low	High	Low	Low
Other biases	Low	Low	Low	Not	Not	Low	Not	Not	Not	Low	Low	High	Not	Low

Note: In this table, “Low” stands for low risk, “High” stands for high risk, and “Not” stands for not clear

### Results of meta-analysis

#### Meta-analysis about VAS score

**Fig. 2:**
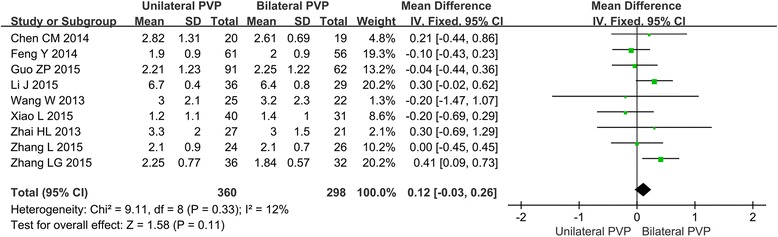
Forest plot for the VAS score in unilateral and bilateral PVP groups

Nine of the 14 included studies report on VAS score for analgesic efficacy evaluation. The forest plot for the VAS score in unilateral and bilateral PVP groups is shown in Fig. 2. Among these 9 articles, only Zhang LG’s study showed the statistical difference between unilateral and bilateral PVP (MD = 0.41, 95%CI [0.09, 0.73]). The other studies showed no statistical significance. The meta-analysis suggested that there was no significant difference of the VAS score in the unilateral group and bilateral group (MD = 0.12, 95%CI [−0.03, 0.26], *P* = 0.11; *P* for heterogeneity = 0.33, *I* ^2^ = 12%).

#### Meta-analysis about ODI score

**Fig. 3:**
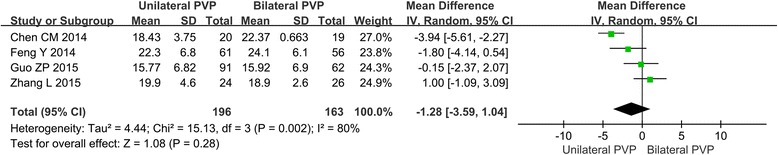
Forest plot for the ODI score in unilateral and bilateral PVP groups

Four included studies report on the ODI score between unilateral and bilateral PVP groups. As shown in the forest plot (Fig. 3), Chen CM reported the statistical difference between unilateral and bilateral PVP (MD = −3.94, 95%CI [−5.61, −2.27]). The result of the meta-analysis showed that the difference of ODI score between unilateral and bilateral PVP was not significant (MD = −1.28, 95%CI [−3.59, 1.04], *P* = 0.28; *P* for heterogeneity = 0.002, *I* ^2^ = 80%).

#### Meta-analysis about the surgery time

**Fig. 4:**
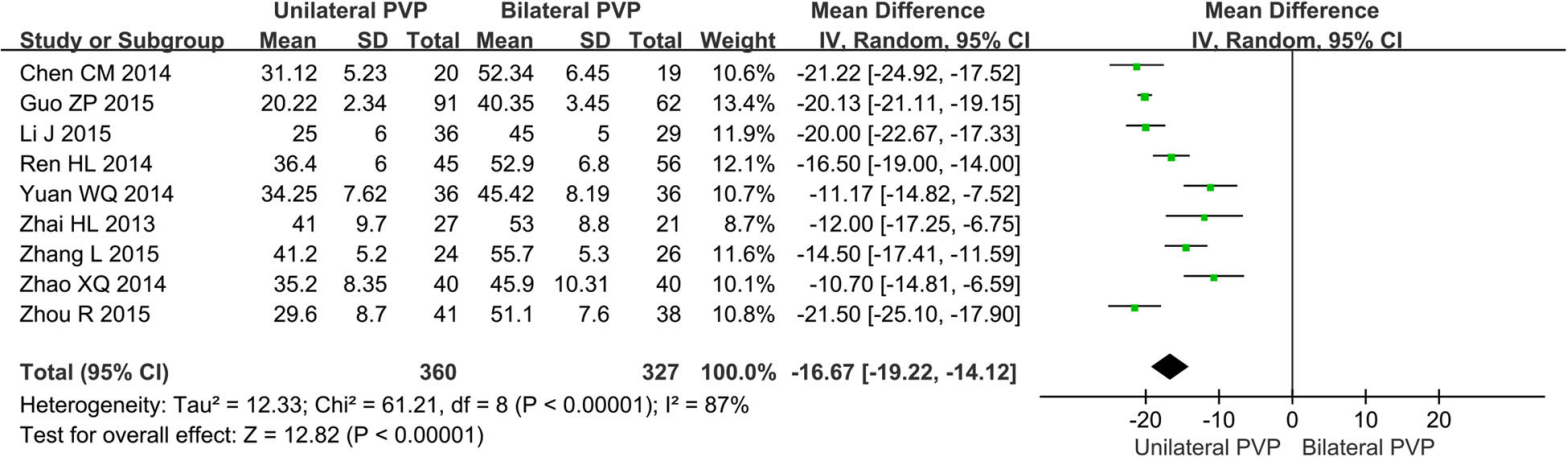
Forest plot for the surgery time in unilateral and bilateral PVP groups

Among the 14 articles, there are 9 studies which are about the comparison of surgery time. The forest plot for the surgery time is shown in Fig. 4. All these 9 studies showed the significant differences of surgery time between unilateral and bilateral PVP, and the meta-analysis indicated that the surgery time of unilateral PVP is much less than that of bilateral PVP (MD = −16.67, 95%CI [−19.22, −14.12], *P* < 0.00001; *P* for heterogeneity < 0.00001, *I* ^2^ = 87%).

#### Meta-analysis about the injected cement volume

**Fig. 5:**
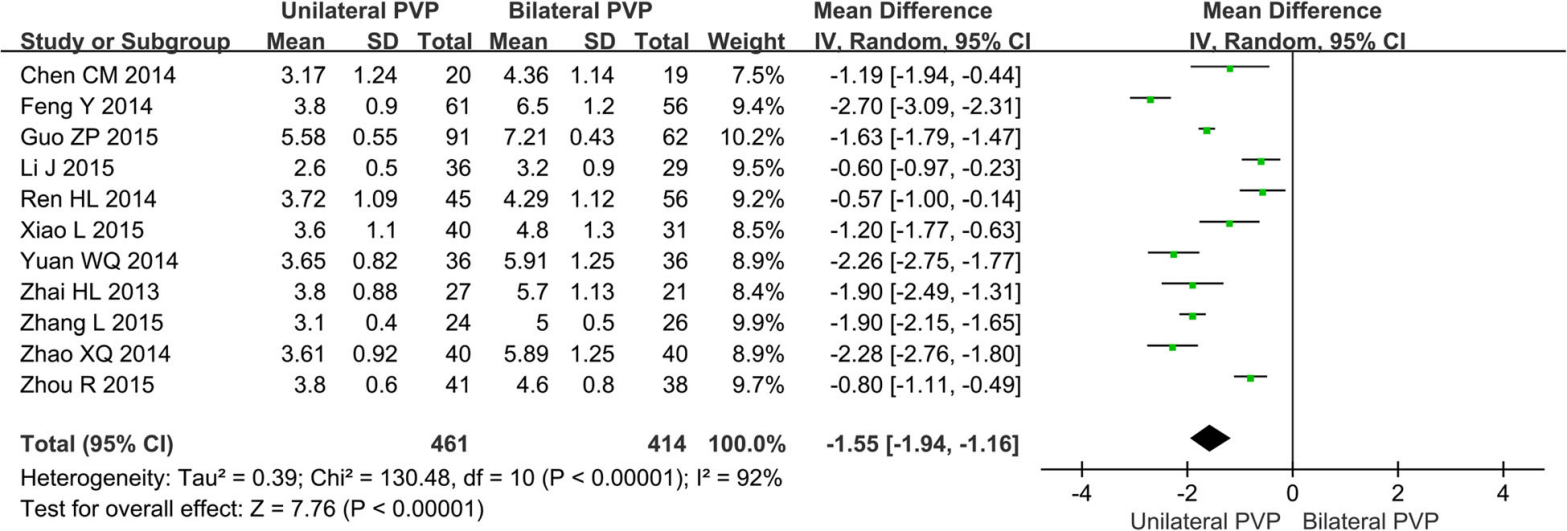
Forest plot for the injected cement volume in unilateral and bilateral PVP groups

All the 11 studies on the cement volume injected in PVP have showed the statistically significant difference between unilateral and bilateral PVP. The result of the meta-analysis indicated that in bilateral PVP surgery, patients with OVCFs have been injected more cement than patients in a unilateral surgery (MD = −1.55, 95%CI [−1.94, −1.16], *P* < 0.00001; *P* for heterogeneity < 0.00001, *I* ^2^ = 92 %). The forest plot for the injected cement volume is shown in Fig. 5.

#### Meta-analysis about the cement leakage rate

**Fig. 6:**
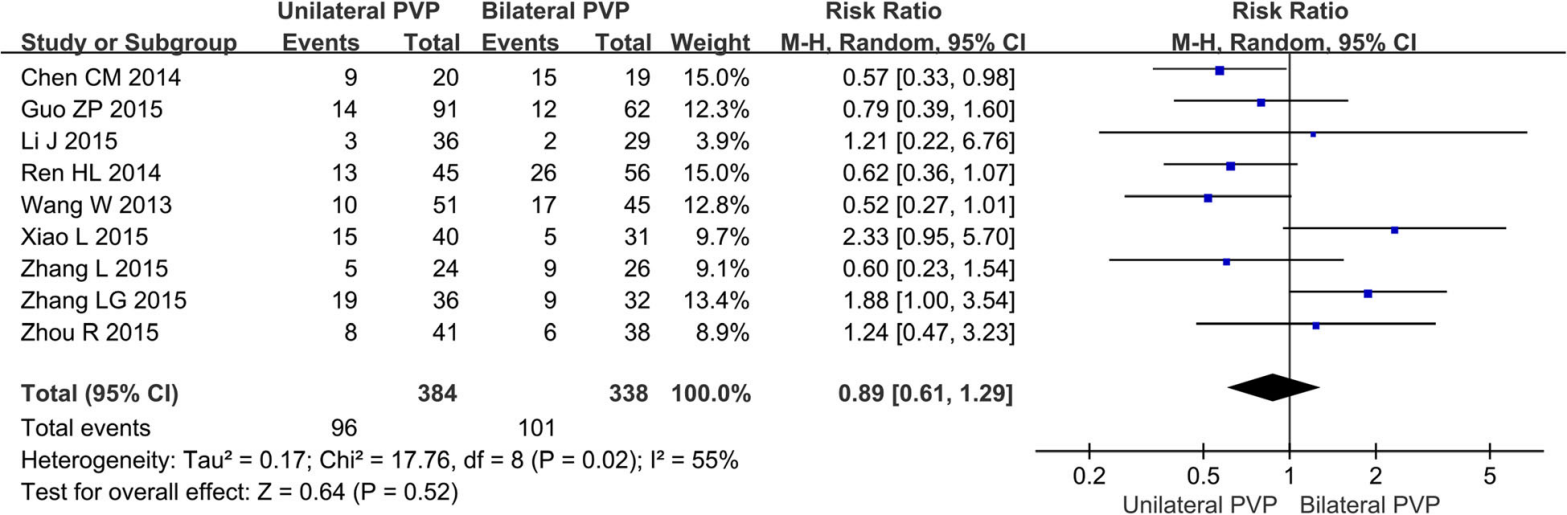
Forest plot for the cement leakage outcome in unilateral and bilateral PVP groups

Among the 14 included studies, 11 are about the cement leakage outcome in unilateral and bilateral PVP groups. The forest plot (Fig. 6) showed that only Chen CM has reported the statistical difference of cement leakage rate between unilateral and bilateral PVP (RR = 0.57, 95%CI [0.33, 0.98]). The result of the meta-analysis showed that the difference of cement leakage rate between unilateral and bilateral PVP was not significant (RR = 0.89, 95%CI [0.61, 1.29], *P* = 0.52; *P* for heterogeneity = 0.02, *I* ^2^ = 55%).

### Bias analysis

According to the results above, high heterogeneities of the ODI score, surgery time, injected cement volume, and cement leakage rate were observed (*I* ^2^ = 80, 87, 92, and 55%, respectively).

**Fig. 7:**
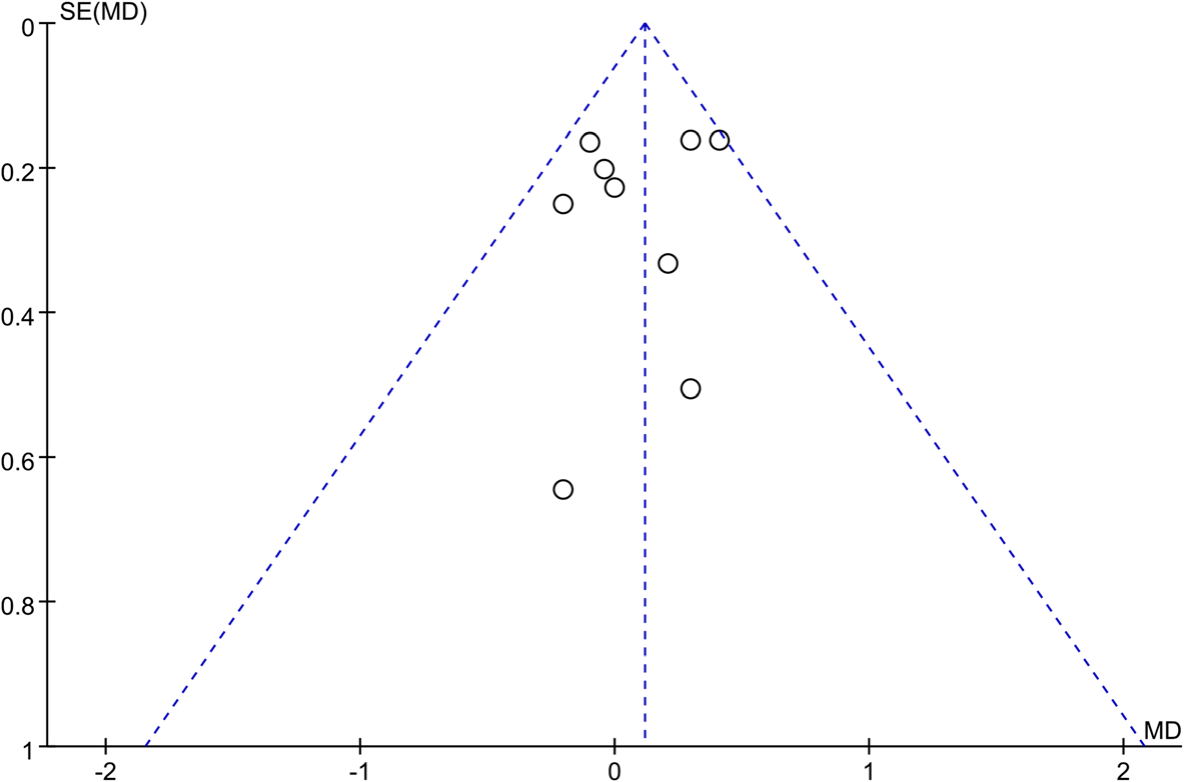
Funnel plot for the studies about VAS score in unilateral and bilateral PVP groups

A funnel plot for the studies about VAS score in unilateral and bilateral PVP groups was performed (Fig. 7). Egger's tests of different parameters are presented in Table 3, which showed that no publication bias was observed in these meta-analyses (*P* > 0.05).

**Table 3:** Egger’s tests of different parameters

	Coefficient	Standard error	*t*	*P* > |*t*|	[95% confidence interval]
VAS score	−0.612	1.017	−0.600	0.566	[−3.016648, 1.792176]
ODI score	10.550	7.103	1.490	0.276	[−20.01237, 41.11181]
Surgery time	3.177	1.427	2.230	0.061	[−0.1973189, 6.552231]
Injected cement volume	0.445	2.572	0.170	0.867	[−5.372733, 6.262107]
Cement leakage rate	0.206	0.181	1.140	0.293	[−0.2219323, 0.6332165]

## Discussion

Excellent strategies for OVCFs are treatments with effective management of pain, short time of recovery, and no requirement of an extended nursing and rehabilitation care [37]. Longo et al. [38] had reported the evidence available on the conservative care for patients with OVCFs and focused on the role of the most commonly used spinal orthoses. But conservative management has not been standardized. Since percutaneous vertebroplasty was introduced more than 30 years ago, researchers extended to vertebral fractures. In the past decades, it has been proved that PVP is a safe and effective surgery. In these studies [17–20], both unipedicular and bipedicular surgeries achieved satisfactory results and patients’ clinical outcome parameters were significantly improved and consistent compared to pre-surgical condition. Some have proved that compared with bilateral PVP, unilateral PVP surgery provides comparable restoration of vertebral body stiffness and therapeutic effects [39, 40]. In this meta-analysis, we compared the effectiveness between unilateral and bilateral PVP through VAS, ODI, surgery time, injected cement volume, and cement leakage outcome.

Owing to the cytotoxic effect of polymethylmethacrylate (PMMA), which is injected into the bones and causes damage to terminal nerve endings, and the immobility and inhibition of micro movement in the fractured fragment [41, 42], significant pain reduction was achieved in the PVP groups who suffered from thoracolumbar compression fractures [43]. VAS is a psychometric response scale used in questionnaires, which measures the subjective characteristics or attitudes that cannot be directly measured. Studies use VAS to estimate pain relief after PVP surgery compared with pre-operation. The meta-analysis suggested that there was no significant difference of the VAS score in the unilateral group and bilateral group, which means that the pain relief of the unilateral group is as much as that of the bilateral group. ODI is an index used by clinicians and researchers to quantify disability for low back pain. A score of 0 is equated with no disability and 100 is the maximum disability possible. The results showed that the ODI score of unilateral PVP was similar to that of bilateral PVP. Also, as we had expected, the bilateral surgery costs more time and more material than the unilateral surgery.

Although relatively safe and effective, PVP may cause complications including cement leakage, soft tissue damage, pedicle fracture, nerve injury, and spinal epidural hematoma [44]. Cement leakage is one of the most frequent complications of vertebroplasty. Symptoms of nerve irritation through compression of nerve roots may be caused by the leakage. It has been reported that pulmonary cement embolism (PCE) following vertebroplasty existed. Cement leakages of both unilateral and bilateral PVP are reported to be as high as 73% [9], but most leakages remain clinically asymptomatic, and even small quantities of leakage may have a significant clinical impact, which is often recognized by their clinical signs and symptoms such as chest pain, dyspnea, tachypnea, coughing, and sweating [45]. In this study, the total cement leakage rates of both groups were 25% (96/384) and 29.88% (101/338), respectively, but no statistically significant difference was observed. The results suggested that increased bone cement injection did not result in increased bone cement leakage rate, and it may be attributed to the nature of the high-viscosity bone cement itself.

Our results support the point that the unipedicular technique is a faster, lower risk alternative that provides a comparable spinal deformity correction than the bipedicular approach. It also increased the cost-effectiveness of the procedure for injecting less cement and cost less surgery time, which lowers the risk of surgery and morbidity.

However, this study has some limitations. In this study, we chose the random-effect model for high heterogeneities of the meta-analyses (*I* ^2^ > 50%). The reasons for high heterogeneities were complex, including different surgical technologies used, varying types of fractures, pre-surgical medical status, and different follow-up times. Also non-RCT studies cause greater bias risks than RCT studies. Besides, the poor quality of some studies indicates that further well-designed and prospective studies are needed to determine the clinical outcomes, which this study did not cover.

## Conclusions

Both unipedicular and bipedicular surgery are safe and effective treatments for OVCF. Although both punctures provide excellent pain relief and improvement of life quality, we encourage the use of the unipedicular approach as the preferred surgical technique for treatment of OVCFs due to less operation time, limited X-ray exposure, and minimal cement introduction and extravasation.

## Abbreviations

OVCFs: Osteoporotic vertebral compression fractures
OVFs: Osteoporotic vertebral fractures
PVP: Percutaneous vertebroplasty
RR: Related risk
SMD: Standard mean difference
VAS: Visual analog scale
